# The Effect of Fava Bean (*Vicia faba* L.) Protein Ingestion on Myofibrillar Protein Synthesis at Rest and after Resistance Exercise in Healthy, Young Men and Women: A Randomised Control Trial

**DOI:** 10.3390/nu14183688

**Published:** 2022-09-06

**Authors:** Robert W. Davies, Marta Kozior, Arthur E. Lynch, Joseph J. Bass, Philip J. Atherton, Ken Smith, Philip M. Jakeman

**Affiliations:** 1 Department of Physical Education and Sport Sciences, University of Limerick, V94 T9PX Limerick, Ireland; 2 Chester Medical School, University of Chester, Shrewsbury SY3 8HQ, UK; 3 Health Research Institute, University of Limerick, V94 T9PX Limerick, Ireland; 4 Centre of Metabolism, Ageing and Physiology, School of Medicine, University of Nottingham, Derby DE22 2DT, UK; 5 MRC–Versus Arthritis Centre for Musculoskeletal Ageing Research and NIHR Nottingham Biomedical Research Centre, School of Medicine, University of Nottingham, Derby DE22 3DT, UK

**Keywords:** amino acids, dietary proteins, deuterium, exercise, muscle mass, plant proteins, resistance training, skeletal muscle, vegetarian, vicia faba

## Abstract

The aim of the present study was to evaluate the effect of feeding fava bean (*Vicia faba* L.) protein (FBP) on resting and post-exercise myofibrillar fractional synthetic rate (myoFSR). In a parallel, double-blind, randomised control trial, sixteen young, healthy recreationally active adults (age = 25 (5) years, body mass = 70 (15) kg, stature = 1.72 (0.11) m, mean (SD)) ingested 0.33 g·kg^−1^ FBP (*n* = 8) or a negative control (CON, i.e., EAA-free mixture) (*n* = 8), immediately after a bout of unilateral knee-extensor resistance exercise. Plasma, saliva, and m. *vastus lateralis* muscle samples were obtained pre-ingestion and 3 h post-ingestion. MyoFSR was calculated via deuterium labelling of myofibrillar-bound alanine, measured by gas chromatography–pyrolysis–isotope ratio mass spectrometry (GC-Pyr-IRMS). Resistance exercise increased myoFSR (*p* = 0.012). However, ingestion of FBP did not evoke an increase in resting (FBP 29 [−5, 63] vs. CON 12 [−25, 49]%, *p* = 0.409, mean % change [95% CI]) or post-exercise (FBP 78 [33, 123]% vs. CON 58 [9, 107]%, *p* = 0.732) myoFSR. Ingestion of 0.33 g·kg^−1^ of FBP does not appear to enhance resting or post-exercise myoFSR in young, healthy, recreationally active adults.

## 1. Introduction

Physiological regulation of skeletal muscle mass is principally determined by the rate of muscle protein synthesis (MPS), specifically the myofibrillar fractional synthetic rate (myoFSR), which can be upregulated by ingestion of dietary protein and contractile activity [1]. Acute ingestion of dietary protein stimulates MPS in a dose-dependent manner [2], driven by an increase in circulating essential amino acids (EAA), particularly leucine, leading to activation of mechanistic target of rapamycin complex 1 (mTORC1) [1]. Contractile activity, such as resistance exercise training (RET), also stimulates MPS via mTORC1 [1]. However, RET can act synergistically with protein ingestion to sensitise skeletal muscle to an increase in postprandial EAA, leading to a greater increase in MPS than can be attained by protein ingestion or RET alone [1,2].

Protein quality (i.e., EAA content, digestibility, and absorption) and the anabolic effect of dietary proteins vary by source [3,4]. Since all EAAs are required as precursors for MPS, research investigating postprandial regulation of MPS has focused on high-quality animal-sourced proteins (e.g., milk proteins) [3], whereas plant-sourced protein is considered to have lower anabolic potency (i.e., ability to stimulate MPS at an equivalent dose) [4]—due to a deficiency in one or more EAAs (e.g., leucine, isoleucine, valine, lysine, methionine and/or tryptophan) and/or lower bioavailability [4]. However, the overall quality of plant-sourced proteins can be improved via commercial processing techniques (i.e., protein extraction and purification) and feeding strategies (e.g., protein complementation) [4,5].

Recently, in response to ongoing environmental, ethical, economic, and health issues, global food directives have promulgated a shift from animal-based to plant-based diets [4]. Successful implementation of these directives is informed by the interrelated outcome of current research on the relative bio-efficacy of plant-based diets and consumer behaviour [4]. Yet, despite great diversity in commercially cultivated plant species, few studies have assessed the potential of commercially processed plant-sourced proteins to stimulate MPS (exceptions being corn [6], wheat [7], potato [8], and soy [9]). *Viciafaba* L. is a cheap, agronomic, and environmentally friendly member of the Fabaceae (legume) family—commonly known as fava, faba, horse, field, or broad bean [10,11]. It is protein-dense (approx. 25% total mass) and lysine-rich (approx. 7% protein mass) [10,11]. Using proprietary methods to extract and remove non- and anti-nutritional factors, concentrated fava bean protein (>70% protein by mass) could act as a potential alternative to animal-sourced protein.

The purpose of the present study was to examine the effect of fava bean protein (FBP) ingestion on resting and post-RET myoFSR in healthy young men and women. We hypothesised that a 0.33 g·kg^−1^ bolus of FBP would stimulate an increase in the myoFSR at rest (REST) and following RET. To test our hypothesis, we used a randomised control parallel group design (FBP vs. CON), unilateral knee extensor RET (REST vs. RET), and contemporary stable isotope (deuterated water) methodology to measure basal, postprandial, and post-exercise myoFSR. An isonitrogenous, non-bioactive mixture of nonessential amino acids only was employed as the control (CON) [12,13]. The study was pre-registered at https://www.clinicaltrials.gov (identifier: NCT05020808) (accessed on 3 September 2022).

## 2. Materials and Methods

### 2.1. Ethical Statement

The study conformed to the standard set by the Declaration of Helsinki, approved by the University of Limerick Education and Health Sciences Research Ethics Committee (2020_04_07_EHS). Participants were informed of the risks and benefits associated with participation before providing written, informed consent.

### 2.2. Participants

**Figure 1:**
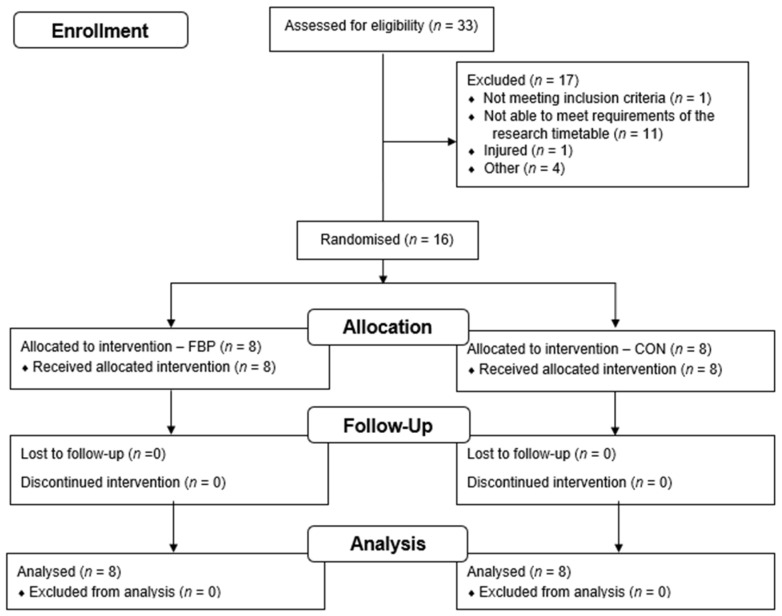
CONSORT flow diagram.

Eligibility criteria were set as: (i) men and women aged from 18 to 35 years; (ii) recreationally active, and (iii) healthy (i.e., not presenting with injury, illness, medication, history of chronic disease, or known allergies/intolerance to the ingredients contained in either formulation, normotensive, non-obese, normal blood chemistry). Sixteen participants were recruited to a parallel-group, double-blind, randomised control trial (age = 25 (5) years, body mass = 70 (15) kg, stature = 1.72 (0.11) m, *n* = 8 per group)—completing the study conduct in full (see Consolidated Standards of Reporting Trials (CONSORT) flow diagram for further details regarding recruitment—Figure 1). Random allocation was performed using random sequences generated by computer software (Microsoft Excel, Microsoft, Redmond, WA, USA) with a block size of 8 per group. Allocation, enrollment, and group assignment were conducted by a separate researcher who was not involved in the conduct, collection, or analysis of study data.

### 2.3. Preliminary Testing

**Figure 2:**
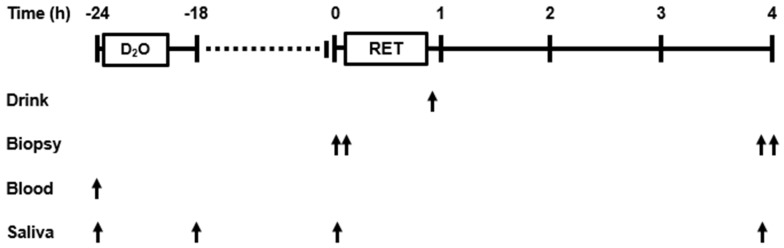
Study design. D_2_O, deuterium oxide bolus (5 mL·kg^−1^ ingested at 2.5 mL·kg^−1^·h^−1^); RET, unilateral knee extensor resistance exercise training; post-exercise beverage was either 0.33 g·kg^−1^ fava bean protein or nonessential amino acids. Arrows indicate time of occurrence.

Participants underwent an initial screening session to assess eligibility. Height (Stadiometer, Seca, Birmingham, UK), weight (Tanita MC-180MA, Tanita Ltd., London, UK), body composition assessment (via a dual-energy x-ray absorptiometry [DXA, Lunar iDXA™, GE Healthcare]), health screen (by a qualified clinician), an exercise familiarisation session, and a 7-day weighed record of habitual dietary and physical activity (i.e., dietary intake, feeding pattern, exercise, and activities of daily living) were completed at least 7 d before starting the experimental trial. Dietary records were analysed by a qualified dietician using Nutritics^®^ software (v.5.7 Research Edition, Nutritics Ltd., Dublin, Ireland). Participants refrained from sports, strenuous physical activity, dietary supplementation, and alcohol consumption for 72 h before the experimental trial. The day before the experimental trial, participants provided saliva and venous blood samples before ingesting 5 mL·kg^−1^ (2.5 mL·kg^−1^·h^−1^) deuterium oxide (D_2_O, 70 atom%) and provided a further saliva sample 2 h later (Figure 2). Venous blood was centrifuged (3500 rpm for 10 min at 20 °C), and aliquots of plasma were frozen at −80 °C. Saliva was centrifuged (13,000 rpm for 10 min at 4 °C), and aliquots of the supernatant were frozen at −80 °C.

### 2.4. Experimental Protocol

A schematic depiction of the experimental protocol is presented in Figure 2. Briefly, participants reported to the University of Limerick human research laboratory between 07:30 to 09:30 fasted overnight (~10 h post-absorptive). Participants provided a saliva sample and bilateral limb micro-biopsy (m. *vastus lateralis*, as described in [14]) to determine basal myofibrillar fraction synthetic rate (myoFSR). Muscle samples were rapidly dissected free of fat and connective tissue, washed in ice-cold saline, snap frozen in liquid nitrogen, and stored at −80 °C. Following bilateral micro-biopsy (~30 min), participants completed a unilateral (dominant limb) knee extension RET session. Immediately post-exercise, participants ingested 0.33 g·kg^−1^ of FBP or CON beverage.

Table 1 contains the amino acid composition of the FBP and CON that were mixed with water (1:10 mass: volume ratio) and administered double-blind. Three hours post-ingestion, a second bilateral micro-biopsy was obtained. During the 3-hour period between biopsies, participants remained seated in the laboratory, and were permitted to only consume water ad libitum.

### 2.5. Resistance Exercise Training Session

The RET was based on a protocol from another study that reported hypertrophy of the quadriceps muscle in young, healthy adults [15]. Briefly, participants completed a familiarisation series of maximal isokinetic contractions (Con-Trex MJ; CMV AG, Dübendorf, Switzerland) of the knee extensors ≥ 7 days before the experimental trial. The RET consisted of 6 sets of 10 maximal effort unilateral (dominant limb) isokinetic knee extensor contractions (concentric and eccentric) at a velocity of 90°·s^−1^. Each set was separated by a 3-minute rest period. Before starting the RET session, participants completed 20 sub-maximal ‘warm-up’ contractions progressively increasing from 50 to 90% of their perceived maximum effort. A Borg (CR10) rating of perceived exertion (RPE) was measured after each set to gauge the participants’ self-reported assessment of effort to ensure they were performing maximal voluntary contractions during each set.

### 2.6. Body Water Enrichment

Precursor enrichment of body water was assessed by heating 100 µL of saliva before being condensed and transferred to an autosampler vial ready for injection into a high-temperature conversion elemental analyser (TC-EA) (Thermo Finnigan, Thermo Scientific, Hemel Hempstead, UK) connected to an isotope ratio mass spectrometer (IRMS) (Delta V Advantage, Thermo Scientific). To minimise the carryover between samples, each sample was injected four times, with the average of the last three injections used for analysis. For accuracy, a standard curve of known D_2_O enrichment was run alongside samples.

### 2.7. Muscle Analysis

Measurement of deuterium labelling of myofibrillar protein-bound alanine was undertaken as previously described [13]. Briefly, 25 mg of muscle was homogenised in ice-cold homogenisation buffer, vortexed for 10 min, and centrifuged at 13,000× *g* for 10 min at 4 °C before the supernatant was removed. The pellet was solubilised in 0.3 M NaOH before centrifugation at 13,000× *g* for 10 min at 4 °C to separate the insoluble collagen fraction. The myofibrillar containing supernatant was subsequently collected and the proteins were precipitated by the addition of 1 M perchloric acid (PCA). For the plasma proteins, 100 µL of the sample was precipitated using 100 µL ice cold ethanol and then separated through centrifugation. Protein-bound amino acids were hydrolysed overnight in 0.1 M HCl and Dowex H+ resin at 110 °C, before elution with 2 M NH4OH and evaporated to dryness. Amino acids were derivatised to their n-methoxycarbonyl methyl esters by resuspension in 60 µL distilled water and 32 µL methanol before vortexing and the addition of 10 µL pyridine and 8 µL methylchloroformate. Samples were further vortexed and extracted in 100 µL chloroform and the addition of a molecular sieve to remove any remaining water before being transferred into a new small volume chromatography vial insert. The deuterium enrichment of protein-bound alanine was measured by sample injection and assessment by gas chromatography–pyrolysis–isotope ratio mass spectrometry (GC-Pyr-IRMS, Delta V Advantage, Thermo Scientific). Samples were injected in triplicate alongside a standard curve of known L-alanine-2,3,3,3-d4 enrichment.

### 2.8. Calculations

MyoFSR was calculated from the incorporation of deuterium-labelled alanine into the myofibrillar protein using body water as a surrogate for precursor enrichment (corrected for the mean number of deuterium moieties incorporated per alanine (3.7) and the total number of hydrogen atoms within the alanine derivative (11)) The equation used is shown below.

(1) $$\mathrm{myoFSR}=-\ln\left(\frac{1-\left[\frac{{\mathrm{APE}}_{\mathrm{Ala}}}{{\mathrm{APE}}_{p}}\right]}{t}\right)$$

where APE_ala_ is deuterium enrichment of protein-bound alanine, APE_p_ is mean precursor enrichment over the study, and *t* is the time between biopsies.

### 2.9. Statistical Analysis

Descriptive statistics are mean (SD). Change (Δ) and percent change (Δ%) values (pre-test to post-test) are reported as mean (95% Student’s-t CI). For statistical analysis, normality and homogeneity of variance were confirmed before performing parametric statistical tests. Mixed-model ANOVA was used to assess group × time and limb × time interactions. Paired *t*-tests were used to assess differences pre-test to post-test within-group, and independent *t*-tests were used to assess between-group differences. The critical significance level was α = 0.05. The magnitude of the change was examined by effect size (i.e., Cohen’s d (d) [16]). Sample size estimates were derived from previously published data (1 − β = 0.8) [2]. Statistical tests were performed in SPSS (v.28, IBM, Armonk, New York, USA), and figures were constructed in Microsoft Excel (Microsoft, Redmond, WA, USA).

## 3. Results

### 3.1. Participant Characteristics

Participant characteristics are provided in Table 2 There were no differences between groups at baseline for any variable reported in Table 2 (*p* > 0.446). During the RET session, participants completed, on average, 16 (6) kJ of work and were able to maintain high-power outputs throughout the RET session (average power output = 130 (50) W). There was no difference in total work conducted (kJ), power output (W), eccentric and/or concentric torque output (N·m), or the rate of fatigue (ΔW_time_) in either absolute or relative (per kg body mass) terms, between groups (*p* > 0.333).

### 3.2. Resting Limb

**Figure 3:**
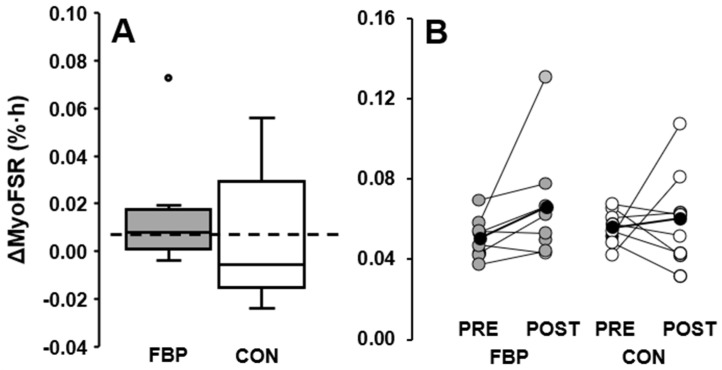
Resting postprandial myofibrillar fractional synthetic rate (myoFSR) change (%·h^−1^). (**A**), change values (post-test pre-test) boxplot; statistical outlier (1.5 × interquartile range, grey circle). (**B**), pre-test (PRE) to post-test (POST) values; data are individual participants from fava bean protein (FBP, grey circles), control (CON, white circles) groups; group means (black circles).

There was no difference between FBP and CON (Group × Time Interaction: *p* = 0.409). MyoFSR did not increase in response to ingestion of either beverage (CON, *p* = 0.670, d = 0.2 [−0.5, 0.9] vs. FBP, *p* = 0.115, d = 0.6 [−0.1, 1.4]) (Table 3 and Figure 3).

### 3.3. Exercised Limb

**Figure 4:**
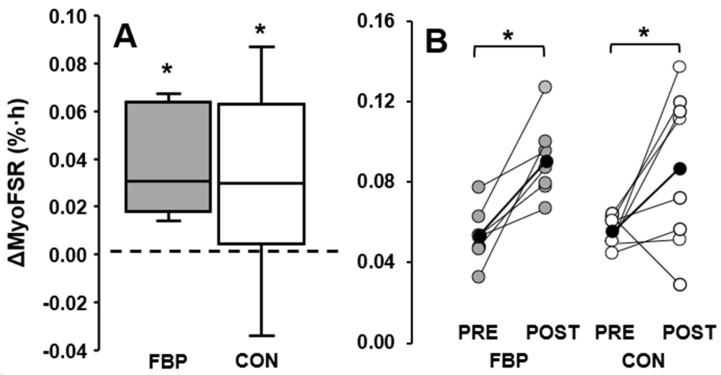
Post-exercise postprandial myofibrillar fractional synthetic rate (myoFSR) change (%·h^−1^) for fava bean protein (FBP) and control (CON) groups. (**A**), change values (post-test – pre-test) boxplot. (**B**), pre-test (PRE) to post-test (POST) values; data are individual participants from fava bean protein (FBP, grey circles), control (CON, white circles) groups; group means (black circles). * Indicates a change from PRE (*p* < 0.05).

There was no difference between FBP and CON (Group × Time Interaction: *p* = 0.732). (Table 3 and Figure 4). However, myoFSR increases were observed within both groups (Time Main Effect: CON, *p* = 0.031, d = 0.8 [0.1, 1.6] vs. FBP, *p* = 0.004, d = 1.7 [0.5, 3.0]) because of contractile activity during RET (Limb × Time Interaction: *p* = 0.012, *n* = 16) (Table 3).

## 4. Discussion

Despite great diversity in crop species, research assessing the effects of plant-sourced proteins on postprandial MPS is limited to just a handful of studies and four commercially cultivated crop species (i.e., corn, soy, potato, and wheat) [6,7,8,9,17]. Therefore, the primary objective of this study was to determine the effect of FBP ingestion on postprandial myoFSR at rest and following RET in healthy, recreationally active young men and women. The response to FBP ingestion was contrasted against a control comprised of an equivalent dose of nonessential amino acids that do not stimulate MPS [12,13]. Results from the present study indicate that 0.33 g·kg^−1^ of FBP does not enhance resting or post-RET myoFSR.

Compared to animal-sourced proteins, the conferred environmental, ethical, and economic benefits of plant-sourced proteins are often mitigated by their lower quality and anabolic potency [4,17]. However, it has been suggested that commercial extraction of protein fraction and removal of bioactive non- and anti-nutritional factors can enhance the anabolic potential [4,5]. Results from the present study do not support this to be the case, as 0.33 g·kg^−1^ of FBP failed to stimulate an acute postprandial (i.e., 3-h) increase in the myoFSR in young, healthy adults.Whereas previous work from our research group has shown that 0.33 g·kg^−1^ of whey protein was effective at stimulating apost-RET myoFSR increase in a group of young, healthy adults over the same time period [13]. Moreover, others have also reported that equivalent, or lower, doses of animal-sourced protein (e.g., whey protein) enhanced, resting or post-RET, postprandial myoFSR [2,7,9].

Since all EAA are required for MPS, the absence of any effect of the FBP could be attributed to an inadequate amount of one or more EAAs [3]. Indeed, analysis of the EAA content of the FBP showed that it contains low levels of methionine (2 mg per g protein) compared to animal-sourced proteins (e.g., 21 mg per g protein in whey protein) [13]. However, it has been shown that acute postprandial increases in the myoFSR (i.e., under 4 h) can be evoked without an exogenous supply of all nine EAA. For example, ingestion of 3.42 g of free leucine [18], 5.6 g of three branched-chain amino acids [19], 30 g of lysine-deficient cereal protein [6,20], and 40 g of methionine deficient soy protein [9] all evoked measurable increases in postprandial myoFSR. This is thought to occur through the depletion of endogenous stores of EAA, acutely supporting higher increases in the myoFSR following anabolic stimulation [21].

A subsequent study from our research group (unpublished observation), in a cohort of nine young, healthy men, revealed that circulating (arterialised) leucine concentrations following ingestion of 0.33 g·kg^−1^ FBP (containing 0.027 g·kg^−1^ leucine or ~2.2 g) were lower than expected (Cmax = 249 (20) μmol·L^−1^)—normally more than 300 μmol·L^−1^ [2,7,19,20]. Therefore, we speculate the absence of any myoFSR increase may have been due to the low bioavailability of leucine following ingestion of the FBP. Indeed, absence of any increase in postprandial myoFSR has been previously reported following ingestion of 35 g of wheat protein (containing 2.5 g leucine) and 20 g of soy protein (containing 2 g leucine), which coincided with lower circulating leucine concentrations (i.e., 200 to 300 μmol·L^−1^) [7,9]. However, both studies also demonstrated that simply increasing the protein dose, and thus leucine (60 g wheat protein and 40 g soy protein, respectively) increased leucinemia and subsequently evoked measurable increases in the myoFSR [7,9]. We speculate that we would see comparable outcomes here (i.e., an increase in the myoFSR) if we were to increase the FBP dose. However, this approach seems redundant from an ecological, economic, practical, and, arguably, health perspective—given proof-of-concept has already been established [7,9]. Alternative strategies such as fortifying or blending lower-quality plant proteins with leucine/EAA or high-quality leucine/EAA-rich proteins [22], improving the bioavailability of leucine/EAA via innovative processing techniques [4], and other technical advancements (e.g., genetic modification increasing leucine/EAA content/bioavailability of plant crops) [4] may be more efficacious and ecologically valid.

Despite the absence of any feeding effect for the FBP, RET was shown to be a potent stimulator of MPS—evoking a robust increase in the myoFSR 3-h post-exercise in both the FBP and CON groups. Whilst noteworthy, but far from novel, this finding adds to the extensive literature supporting RET as a potent stimulator of MPS. Additionally, this also empirically demonstrates we have the sensitivity to capture moderate to large changes in the myoFSR over a short period of time (<4 h), using deuterated water methodology, which is usually reserved for diurnal FSR measurement [23,24].

Findings from the present study add to a growing body of evidence, demonstrating that distinctions need to be made between dietary protein sources (e.g., plant vs. animal), as they are not physiologically comparable or interchangeable on a gram-to-gram basis. Additionally, it should be noted that it is not just the leucine content of the protein, or even the postprandial leucinemia, that solely determines postprandial myoFSR. Factors such as age, sex, health, nutritional and training status (and the interactions between them) differentially affect anabolism [25,26]. For example, ingestion of 40 g of soy protein was shown to be ineffective at increasing resting myoFSR in older men, whereas ingestion after RET led to robust increases in the myoFSR [9]. Similarly, sexually dimorphic postprandial MPS rates have been reported in older, but not younger, men and women [25,26,27,28]. Indeed, a novel aspect of the present study was the use of a mixed-sex cohort, whereas most research in this area is limited to single-sex studies (i.e., mostly men). The rationale for including women in this study was to bridge the gap related to sparse evidence in the investigation of the effect of protein intake and RT on myoFSR. However, consideration was given to the potential influence of the menstrual cycle effect on the aim of this study. According to the scientific literature, it has been shown that the menstrual cycle does not affect MPS [29]. Consequently, careful consideration had to be taken in the recruitment, allocation, standardisation, control of the RET, and protein dose between participants. However, despite careful planning and implementation of the study conduct, we still noticed greater heterogeneity within treatment groups compared to some single-sex studies [2,9,13]. In future studies, greater attention should be given to hormonal contraceptives used by women as they are suggested to affect MPS [29]. Here, a larger sample size would be required to rule out that sex directly influenced results in the present study.

## 5. Conclusions

To conclude, ingestion of 0.33 g·kg^−1^ of fava bean (*Vicia faba* L.) protein does not enhance resting or post-exercise myoFSR in young, healthy, and recreationally active men and women, whereas RET was a potent stimulator of MPS. These data provide useful insight for developing and optimising the role that plant-sourced proteins play in stimulating muscle protein synthesis, regulating muscle protein metabolism, and supporting growth, maintenance, and preservation of muscle mass.

## Figures and Tables

**Table 1:** Amino acid composition of the two feeds.

	FBP	CON	FBP	CON
AA Profile	g per 100 g		g per kg Body Mass	
Alanine	3.0	10.7	0.014	0.035
Arginine	6.5	0.0	0.031	0.000
Aspartate	8.4	12.9	0.040	0.043
Cysteine	0.8	0.0	0.004	0.000
Glutamate	12.4	36.4	0.059	0.120
Glycine	2.9	3.9	0.014	0.013
Histidine	1.9	0.0	0.009	0.000
Isoleucine	3.0	0.0	0.014	0.000
Leucine	5.7	0.0	0.027	0.000
Lysine	4.8	0.0	0.023	0.000
Methionine	0.5	0.0	0.002	0.000
Phenylalanine	3.4	0.0	0.016	0.000
Proline	3.2	15.6	0.015	0.051
Serine	3.8	13.5	0.018	0.045
Threonine	2.6	0.0	0.012	0.000
Tryptophan	0.6	0.0	0.003	0.000
Tyrosine	2.5	6.9	0.012	0.023
Valine	3.4	0.0	0.016	0.000
EAA	25.8	0.0	0.123	0.000
TAA	69.3	100	0.330	0.330

FBP, fava bean protein; CON, control; EAA, essential amino acids; TAA, total amino acids.

**Table 2:** Participant baseline characteristics.

	FBP	CON
Group Size	8	8
Sex (M:F)	4:4	4:4
Age (years)	25 (3)	25 (6)
Stature (m)	1.73 (0.11)	1.71 (0.10)
Body Mass (kg)	72 (19)	68 (10)
LTMI (kg·m^−2^)	17 (2)	18 (3)
% Fat Mass	21 (7)	23 (7)
Energy Intake (kcal·kg^−1^·d^−1^)	32 (6)	37 (10)
Protein Intake (g·kg^−1^·d^−1^)	1.4 (0.4)	1.5 (0.5)

Data are mean (SD). FBP, Fava bean protein; CON, control.

**Table 3:** Myofibrillar fractional synthetic rates.

	REST				RET			
	PRE	POST	Δ	Δ%	PRE	POST	Δ	Δ%
FBP	0.050 (0.010)	0.066 (0.020)	0.016 (0.020)	29 (34)	0.054 (0.014)	0.091 (0.020) *	0.037 (0.016) *	78 (45) *
CON	0.056 (0.009)	0.060 (0.024)	0.004 (0.018)	12 (37)	0.055 (0.008)	0.087 (0.039) *	0.031 (0.027) *	58(49) *

Data are mean (SD) %/h. Change data (Δ) are mean (95% CI). * indicates a change from PRE (*p* < 0.05). REST, rested limb; RET, exercised limb; PRE, baseline; POST, 3-h post-ingestion/post-exercise. CON, control; FBP, fava bean protein.

## Data Availability

The data presented in this study are available in Figure 3 and Figure 4.
